# First Insights into the Genome of the Gram-Negative, Endospore-Forming Organism *Sporomusa ovata* Strain H1 DSM 2662

**DOI:** 10.1128/genomeA.00734-13

**Published:** 2013-09-12

**Authors:** Anja Poehlein, Gerhard Gottschalk, Rolf Daniel

**Affiliations:** Genomic and Applied Microbiology and Göttingen Genomics Laboratory, Institute of Microbiology and Genetics, Georg-August University Göttingen, Göttingen, Germany

## Abstract

The genome of *Sporomusa ovata* strain H1 DSM 2662, an anaerobic, Gram-negative endospore-forming bacterium, was sequenced. *S. ovata* uses *N*-methyl compounds, primary alcohols, fatty acids, and H_2_ and CO_2_ as energy and carbon sources to produce acetate. The genome harbors one chromosome, which encodes proteins typical for sporulation.

## GENOME ANNOUNCEMENT

The Gram-negative endospore-forming bacterium *Sporomusa ovata* belongs to the class *Negativicutes* within the *Firmicutes*. This class comprises only a few genera, which are Gram negative and form endospores. *S. ovata* was one of the first described species with this feature (1). Based on genomic comparisons of Gram-negative members of the *Firmicutes*, the assignment of *Sporomusa* to the new family *Sporomusaceae* was recommended (2). *S. ovata* ferments *N*-methyl compounds, such as betaine, *N*,*N*-dimethylglycine, and sarcosine, but also primary alcohols, hydroxy fatty acids, and 2,3-butanediol. The main product is acetate, which is also produced from H_2_ and CO_2_.

Genomic DNA of *S. ovata* strain H1 DSM 2662 was isolated with the MasterPure complete DNA purification kit (Epicenter, Madison, WI). The extracted DNA was used to generate 454-shotgun, paired-end, and Illumina-shotgun libraries according to the manufacturer’s protocols. The libraries were sequenced using a 454 GS-FLX system (Titanium GS70 chemistry; Roche Life Sciences, Mannheim, Germany) and Genome Analyzer II (Illumina, San Diego, CA). Sequencing resulted in coverages of 17.99 and 101.75, respectively, with the two sequencing systems. Assembly of the reads using Roche Newbler assembly software 2.6 for scaffolding and MIRA software (3) resulted in 37 scaffolds with 60 contigs. The remaining gaps were closed with PCR-based techniques and Sanger sequencing of the products (4) employing the Gap4 (v.4.11) software of the Staden package (5). The draft genome of *S. ovata* H1 DSM 2662 comprised one circular chromosome of 5.38 Mb with an overall G+C content of 42.25 mol%. Functional annotation of the 5,110 predicted protein-encoding genes was initially carried out with the IMG/ER (Intergrated Microbial Genomes/Expert Review) system (6, 7). Subsequently, annotations were manually curated by using the Swiss-Prot, TREMBL, and InterPro databases (8). The genome harbored at least 13 rRNA operons and 127 tRNA genes, which were identified with RNAmmer and tRNAscan, respectively (9, 10).

Analysis of the genome sequence revealed the presence of various sensory histidine kinase (KinACDE) transcription and sigma factors such as Spo0A, σ^H^, σ^F^, σ^E^, σ^G^, and σ^K^, which are essential for initiation of sporulation (11, 12). At least 83 genes coding for proteins involved in the various stages of sporulation were identified, and all such proteins were orthologous to known proteins involved in sporulation of *Clostridia* and *Bacilli* (13).

Genes coding for outer membrane proteins, chaperones, and outer membrane efflux proteins were detected, as well as genes for lipid A biosynthesis acetyl transferases and lipid A disaccharide synthetases. In addition, a putative *pylTScBCDSn* gene cluster encoding proteins necessary for incorporation of pyrrolysine into proteins was present (14). Upstream of this cluster, putative genes encoding corrinoid-dependent and pyrrolysine-containing methylamine methyltransferases (15) were located. Besides those in the *Methanosarcinaceae*, in which the *pyl* genes were discovered, we identified these genes by genome comparisons in only a few genera belonging to the *Peptococcaceae*, *Halobacteroidaceae*, and *Thermoanaerobacteriaceae*, which are, as is *S. ovata*, members of the *Firmicutes*.

### Nucleotide sequence accession numbers.

The draft genome sequence of *Sporomusa ovata* H1 DSM 2662 has been deposited at DDBJ/EMBL/GenBank under the accession number ASXP00000000. The version described is version ASXP01000000.
